# Possibilities of Anxiolytic Therapy in the Elimination of Stress Skin Manifestations: A Case Report

**DOI:** 10.15388/Amed.2023.30.1.8

**Published:** 2023-05-16

**Authors:** Nataliia Sydorova, Volodymyr Vereshchaka, Taras Kuts

**Affiliations:** Department of Military General Practice & Family Medicine, Ukrainian Military Medical Academy, 45/1-33 Knyaziv Ostrozʹkykh str., 01015, Kyiv, Ukraine; Educational and Scientific Center “Institute of Biology and Medicine”, Taras Shevchenko National University of Kyiv, 2 Hlushkova Avenue, 03127, Kyiv, Ukraine; Department of Military General Practice & Family Medicine, Ukrainian Military Medical Academy, 45/1-33 Knyaziv Ostrozʹkykh str., 01015, Kyiv, Ukraine

**Keywords:** itching, exanthema, anxiolytic, psychological stress, temgicoluril

## Abstract

The case of a 42-year-old female patient with pronounced itching and exanthema, mainly in the area of the trunk and lower limbs, is presented. Previously, the patient took antihistamines without effect, was treated for scabies, but the itching remained pronounced and led to rash and excoriations. From the anamnesis, it was found that the patient has a high level of stress. According to the Hospital Anxiety and Depression Scale, the anxiety of the patient reached 14 points, and depression 1 point. Functional (psychogenic) itching was suspected. Since the patient refused dermatologist consultation, therapy with the anxiolytic temgicoluril, topical antipruritic agents and nonpharmacological methods of treatment were recommended at the initial stage. The patient felt a significant relief of itching symptoms on the first day of anxiolytic usage, she withdrew topical antipruritic agents after 5 days of anxiolytic treatment, in 15 days she began to reduce the dose of temgicoluril, and at the end of the third week she stopped treatment with anxiolytic due to a significant positive effect. In three weeks, practically all elements of the rash, except for the largest wounds from scratching, disappeared. The peculiarity of the case is that functional itching was completely eliminated during anxiolytic therapy without other systemic medications, which emphasizes the importance of eliminating the component of stress and anxiety in the treatment of such patients.

## Santrauka.

Aprašomuoju atveju 42 metų pacientei buvo nustatyti niežulys ir egzantema ryškiausia liemens ir apatinių galūnių srityje. Anksčiau pacientė vartojo antihistaminus, tačiau jie poveikio nedarė. Pacientė buvo gydoma nuo niežų, tačiau niežulys išliko ryškus, atsirado bėrimų, besikasydama susibraižydavo odą. Anamnezė atskleidė, kad pacientė patirdavo didžiulį stresą. Pagal klinikinę nerimo ir depresijos skalę (*Hospital Anxiety and Depression Scale*) nustatyta, kad pacientės nerimas siekė 14 taškų, o depresija – 1 tašką. Kilo įtarimų, kad pasireiškė funkcinis (psichogeninis) niežulys. Kadangi pacientė atsisakė konsultuotis su dermatologu, pradiniame etape buvo rekomenduotas gydymas anksiolitiniu temgikolurilu, vietiniu antipruritiniu preparatu bei nefarmaciniai metodai. Jau pirmąją anksiolitikų vartojimo dieną pacientei pastebimai susilpnėjo niežulio simptomai, o penktą gydymo anksiolitais dieną ji liovėsi vartoti vietinius antipruritinius preparatus, o po 15 dienų pradėjo mažinti temgikolurilo dozę. Baigiantis trečiai savaitei pacientė nutraukė gydymąsi anksiolitikais, nes buvo pasiektas ryškus teigiamas pokytis. Per tris savaites bėrimas beveik išnyko, išskyrus didžiausias kasymosi sukeltas žaizdas. Šis atvejis išskirtinis, nes taikant anksiolitikų terapiją be jokių kitų sisteminių medikamentų pavyko eliminuoti funkcinį niežulį. Tai atskleidžia, kad, gydant tokius pacientus, svarbu pašalinti streso ir nerimo veiksnį.

*“It is the brain that itches, not the skin” [1].*

## Introduction

The problem of functional (psychogenic) changes in the skin, in particular itching, is important for the practice of a family doctor. However, the number of scientific publications on this problem is limited [2]. Stress and anxiety accompany modern human life and can act as risk factors, triggers or confounders in various diseases, including provoking pathological skin conditions, such as psychogenic pruritis. The development of itching and its consequences in the form of exanthema due to scratching have been described both in acute stress and chronic exposure to this factor [1]. To verify such a condition, which requires the mandatory prior exclusion of all other possible causes of itching, it is recommended to use the diagnostic criteria for functional pruritus (or psychogenic pruritus) from the French Psychodermatology Group (FPDG) [2].

Stress-induced itching can develop due to various pathophysiological mechanisms. Stress can provoke the activation of mast cells through several neurotransmitters (substance P, calcitonin gene-related peptide, etc.) [1,3]. An effect on the function of T and B cells has been described in patients with psychological stress, which can predispose patients to skin manifestations like urticaria or itching [3]. Numerous pruritogenic stimuli, both endogenous and exogenous, have been described, and some of them are also stress-related [1]. A connection was found between increased NLRH-3 inflammasome activity and stress [3,4]. Recently found C-type fibers can also be responsible for itching in patients with psychological stress [1,3]. Itching forms a pathological circle: it leads to scratching, and wounds only increase the sensation of itching [2].

At the same time, patients sometimes refuse to consult a specialist, feel uncomfortable when discussing this problem and believe that it is better to be self-treated at home without publicity. This leads to long-term suffering, increased stress, disturbed sleep, forming a pathological cycle of the problem. One of the important aspects of the management of such patients is the strengthening of trust in the doctor and therapy, which is often achieved through interventions focused on the mental state of the patient: psychotherapy and the use of psychotropic agents [5].

The presented case is of interest because the patient, a doctor herself, avoided a visit to dermatologist, underwent an examination and treatment *ex juvantibus* on her own before consultation with her family doctor. Such behavior, although contrary to recommendations, is quite typical for real clinical practice and should be considered in the work of a family doctor.

## Case Report

A 42-year-old female patient turned to her family doctor for complaints of pronounced itching of the skin and rash as a result of scratching. A symmetrical exanthema in the form of multiple excoriations, crusts, vesicles, and isolated hematomas was found on the trunk and lower extremities.

The patient noted these symptoms for the last 3 weeks before the visit, which were more pronounced in the evening and at night. She suffered from unbearable itching when exposed to hot water while taking a shower. The symptoms led to sleep disturbances and reduced work capacity.

The following is known from the anamnesis: personal allergic history was aggravated (allergy to pet saliva, intolerance to some cosmetics), as well as family one (on the mother`s side), a diagnosis of nonalcoholic fatty liver disease was established against the background of 3rd degree alimentary obesity. Prior to the onset of symptoms, exposure to several potential triggers (cold weather, dry skin, new detergents/cosmetics, consumption of new unfamiliar foods and dietary supplements) has taken place.

The patient underwent a laboratory examination in order to exclude typical somatic causes of itching, in particular blood and liver diseases. There were neither polycythemia nor eosinophilia. Signs of minor cholestasis without negative dynamics for several years were revealed: alanine transaminase 55 U/L, aspartic transaminase 27 U/L, gamma glutamyl transferase 53 U/L, alkaline phosphatase 86 U/L, total bilirubin 4.6 μmol/L. The blood creatinine level was 66 μmol/L, the calculated glomerular filtration rate was 99 mL/min/1.73m^2^.

Based on the most likely causes of her condition, the patient:

- stopped using dietary supplements that may have triggered the symptoms;
- tried antihistamines (loratadine, fexofenadine) for several days without effect, so she eventually stopped the treatment;
- passed a course of deworming with albendazole;
- used antiscabies (permethrin ointment 4%) with little effect and rapid return of symptoms;
- tried therapy aimed at cholestasis, but discontinued it due to increased itching;
- used local anti-itch remedies with virtually no effect.

Since the measures applied by the patient did not bring relief, the areas of excoriations against the background of scratching continued to increase, she decided to consult a family doctor.

During the consultation, the patient repeatedly mentioned stress at work, general nervousness. Assessment by Hospital Anxiety and Depression Scale questionnaire showed anxiety scale score at 14 points (clinically expressed anxiety), depression scale score at 1 point (no depression).

The family doctor offered a dermatologist consultation, which the patient refused due to pessimism and feelings of guilt/discomfort, and additionally:

- wearing loose clothing made of natural cotton, avoiding hot water, applying moisturizing creams immediately after contact with water, applying topical anti-itch remedies as needed;
- for control of excessive stress and anxiety: holistic approaches, physical activity, daytime anxiolytic temgicoluril 500 mg 2 times a day for 3 weeks.

**Figure 1.**
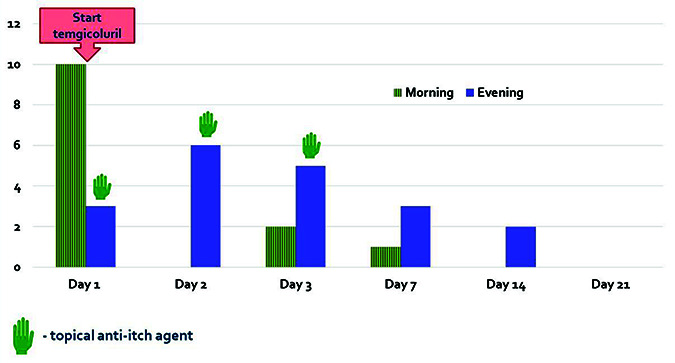
Timeline of the intensity of the patient’s itch after the start of therapy with temgicoluril (additionally only topical anti-itching agent was used as needed) according to visual analogue scale (10 points – the greatest intensity).

The patient felt significant relief after the very first tablet of temgicoluril, the severity of itching decreased more than three times (Figure 1). On the 6th day of temgicoluril therapy, the patient stopped using topical anti-itching agent. On the 15th day, she began to skip the morning doses of temgicoluril. On the 21st day, she stopped taking temgicoluril. Slight skin irritation remained, but the itching stopped. Excoriations were not detected.

**Figure 2.**
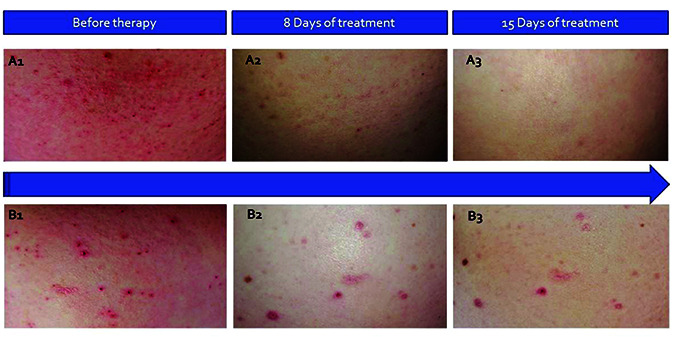
Dynamics of the patient’s rash on the background of temgicoluril therapy. Two sections of the skin of the abdominal wall were provided at the time of consultation (A1 and B1), after 8 days of temgicoluril treatment (A2 and B2), and on the 15th day of the treatment (A3 and B3).

Visually, positive dynamics were also noted in the form of a decrease in the elements of the rash (Figure 2).

It should be noted that the patient’s psychological condition has improved. The patient began to trust the doctor more and finally agreed to consult a dermatologist.

## Discussion

Stress and anxiety are common condition, in particular, among health care providers [6]. Stress rash and itching are generally thought to be the most common in premenopausal women due to certain effects of estrogen and progesterone [3]. Our patient (a woman who is a doctor) actually met FPDG criteria for functional (or psychogenic) pruritus [2].

The role of the family doctor in the management of patients with pruritus includes considering each case in a broad context, excluding its causes related to internal pathology (blood diseases, oncological pathology, cholestasis, etc.) and infectious diseases, referral to specialists, and providing recommendations for nondrug measures to reducing itching.

Diagnosis of psychogenic itching requires not only the exclusion of other causes, but also confirmation of the connection of symptoms with stress and psychological factors [2]. In our case, the criterion that influenced the definition of itching as psychogenic was the pronounced positive effect of treatment with an anxiolytic.

Potential reasons for the ineffectiveness of H_1_-receptor blockers in the presented patient could be as follows:

- incorrectly selected drug, dose (too low), duration of therapy;
- triggers remaining;
- another mechanism – for example, H_4_-receptors have a higher affinity for histamine compared to H_1_-receptors, their activation on T_h_2 cells leads to the release of interleukin 31 and the development of itching.
- functional changes in the central nervous system during stress as well as the hedonic aspects of scratching as a pleasure, which temporarily reduces the feeling of itching, anxiety and stress.

The choice of a psychotropic drug for the patient in order to reduce anxiety was justified by several reasons.

Firstly, this substance was studied in the complex therapy of skin pathologies associated with stress and no pharmacological interactions were observed, which allows safe use with most other remedies [7].

Secondly, it was known about the effect of histamine on the choline, serotonin, glutamate, and adrenergic systems as well as γ-aminobutyric acid (GABA) through H_3_ receptors [8]. E. M. J. Peters notes that: “skin and brain speak the same chemical language” [5]. In turn, temgicoluril is known for its balancing effects on the GABA, choline, serotonin, glutamate, and adrenergic systems [9], and can be an interesting strategy for transforming the process of transmission and processing of the itch sensation in the central nervous system, especially under conditions of stress and anxiety.

It is also interesting that temgicoluril is similar in chemical structure to natural metabolites of the body – its molecule consists of two methylated fragments of urea, which are part of the bicyclic structure [9]. It is known that urea is actively used in dermatology and cosmetology as a substance that promotes skin hydration, has a keratolytic effect and an antipruritic effect [10]. Topical treatment containing urea is indicated for the treatment of skin diseases accompanied by increased formation of keratinized epithelium, in particular psoriasis, seborrhea, hyperkeratoses, hyperkeratic forms of eczema, ichthyosis [10]. There are works that studied at the level of a chemical experiment the interaction between temgicoluril and urea under different conditions and established that temgicoluril exerts a stabilizing effect on the urea hydration process, although the practical significance of these results is currently unknown [11]. However, there are insufficient data from well-designed, controlled clinical trials on the efficacy of temgicoluril as a treatment for stress-related skin problems, and therefore, in our opinion, their planning is reasonable and appropriate.

## Conclusions

1. Psychogenic pruritus and skin manifestations of stress are quite frequent symptoms, as a result of which the patient turns to the family doctor, but the verification of this condition requires the preliminary exclusion of the most common other causes and compliance with the criteria for the diagnosis of functional pruritus proposed by the FPDG.
2. Skin care plays an important role in the treatment of psychogenic itching, and consultation with a dermatologist is mandatory.
3. Therapy with psychotropic drugs, in particular anxiolytics (this case presents therapy with temgicoluril), can significantly improve the condition of the patient with psychogenic pruritus, but well-designed controlled studies are needed to establish this impact.
